# THK5351 and flortaucipir PET with pathological correlation in a Creutzfeldt-Jakob disease patient: a case report

**DOI:** 10.1186/s12883-019-1434-z

**Published:** 2019-08-29

**Authors:** Hee Jin Kim, Hanna Cho, Seongbeom Park, Hyemin Jang, Young Hoon Ryu, Jae Yong Choi, Seung Hwan Moon, Seung Jun Oh, Minyoung Oh, Duk L. Na, Chul Hyoung Lyoo, Eun-Joo Kim, William W. Seeley, Jae Seung Kim, Kyung Chan Choi, Sang Won Seo

**Affiliations:** 1 Department of Neurology, Samsung Medical Center, Sungkyunkwan University School of Medicine, 50 Ilwon-dong, Gangnam-gu, Seoul, 135-710 Republic of Korea; 2 Samsung Alzheimer Research Center, Samsung Medical Center, Seoul, Korea; 3 Neuroscience Center, Samsung Medical Center, Seoul, Korea; 4 Department of Neurology, Gangnam Severance Hospital, Yonsei University College of Medicine, Seoul, Korea; 5 Department of Nuclear Medicine, Gangnam Severance Hospital, Yonsei University College of Medicine, Seoul, South Korea; 6 Division of RI-Convergence Research, Korea Institute of Radiological and Medical Sciences, Seoul, South Korea; 7 Department of Nuclear Medicine, Samsung Medical Center, Sungkyunkwan University School of Medicine, Seoul, Korea; 8 Department of Nuclear Medicine, Asan Medical Center, University of Ulsan College of Medicine, Seoul, Korea; 9 Department of Health Sciences and Technology, SAIHST, Sungkyunkwan University, Seoul, Korea; 10 Department of Neurology, Pusan National University Hospital, Pusan National University School of Medicine and Medical Research Institute, Busan, Republic of Korea; 11 Department of Neurology and Department of Pathology, University of California, San Francisco, CA USA; 12 Department of Pathology, Chuncheon Sacred Heart Hospital, Hallym University College of Medicine, Chuncheon, Korea; 13 Korea CJD Autopsy Center, Hallym University Sacred Heart Hospital, Anyang, Korea; 14 Department of Clinical Research Design and Evaluation, SAIHST, Sungkyunkwan University, Seoul, Korea; 15 Center for Clinical Epidemiology, Samsung Medical Center, Sungkyunkwan University School of Medicine, Seoul, Republic of Korea

**Keywords:** THK5351 PET, Flortaucipir PET, Monoamine oxidase B, Creutzfeldt-Jakob disease

## Abstract

**Background:**

THK5351 and flortaucipir tau ligands have high affinity for paired helical filament tau, yet diverse off-target bindings have been reported. Recent data support the hypothesis that THK5351 binds to monoamine oxidase B (MAO-B) expressed from reactive astrocytes and that flortaucipir has an affinity toward MAO-A and B; however, pathological evidence is lacking. We performed a head-to-head comparison of the two tau ligands in a sporadic Creutzfeldt-Jakob disease (CJD) patient and performed an imaging-pathological correlation study.

**Case presentation:**

A 67-year-old man visited our clinic a history of 6 months of rapidly progressive dementia, visual disturbance, and akinetic mutism. Diffusion-weighted imaging showed cortical diffusion restrictions in the left temporo-parieto-occipital regions. ^18^F-THK5351 PET, but not ^18^F-flortaucipir PET showed high uptake in the left temporo-parieto-occipital regions, largely overlapping with the diffusion restricted areas. Cerebrospinal fluid analysis was weakly positive for 14–3-3 protein and pathogenic prion protein was found. The patient showed rapid cognitive decline along with myoclonic seizures and died 13 months after his first visit. A post-mortem study revealed immunoreactivity for PrP^sc^, no evidence of neurofibrillary tangles, and abundant astrocytosis which was reactive for MAO-B antibody.

**Conclusions:**

Our findings add pathological evidence that increased THK5351 uptake in sporadic CJD patients might be caused by an off-target binding driven by its high affinity for MAO-B.

**Electronic supplementary material:**

The online version of this article (10.1186/s12883-019-1434-z) contains supplementary material, which is available to authorized users.

## Background

THK5351 and flortaucipir tau ligands were developed with the anticipation that they would have specific high affinity for paired helical filament (PHF) type tau, the building blocks of neurofibrillary tangles (NFT) in Alzheimer’s disease. Yet, diverse off-target binding has been reported on positron emission tomography (PET) imaging for each ligand [1]. Accumulating data support the hypothesis that THK5351, a quinolone-derivative agent, binds to monoamine oxidase B (MAO-B) expressed from reactive astrocytes [2]. Flortaucipir has also shown in vitro affinity toward MAO-A and B [3]. In addition, a recent head-to-head comparison of two tau ligands raised the possibility that THK5351 and flortaucipir have distinct characteristics [4].

Sporadic Creutzfeldt-Jakob disease (sCJD), caused by prion protein, is a rapidly progressive neurodegenerative disorder that can have increased MAO-B activity as well as NFT. Previous imaging and pathology studies of CJD showed increased MAO-B expression by activated astrocytes and microglia [5, 6]. Meanwhile, various neuronal and glial tau pathologies exist in sCJD patients, including NFT, and the co-existence of Alzheimer’s disease occurs in 10% of patients with sCJD [7, 8]. Cerebrospinal fluid (CSF) analysis of CJD patients shows highly elevated total tau and slightly elevated phosphorylated tau [9].

In the present study, we performed a head-to-head comparison of two tau (THK5351 and flortaucipir) PET images in a patient with sCJD to find out whether the patient’s brain showed distinct uptake patterns. We further performed an imaging-pathological correlation study to determine whether increased uptake of tau ligands in this case represented increased PHF-type tau burden or MAO-B activity.

## Case presentation

A 67-year-old right-handed man with a history of hypertension visited the Memory Clinic at Samsung Medical Center for rapidly progressive dementia, visual disturbance, and akinetic mutism, which started six months prior to his first visit. On neurologic examination, he showed bilateral bradykinesia, parkinsonian gait, and postural instability. On neuropsychological tests, his Mini-Mental State Examination score was 21 (6 years of formal education). He showed poor performance on language (confrontational naming, comprehension, and repetition tests), visuospatial, memory, and frontal/executive function.

**Fig. 1:**
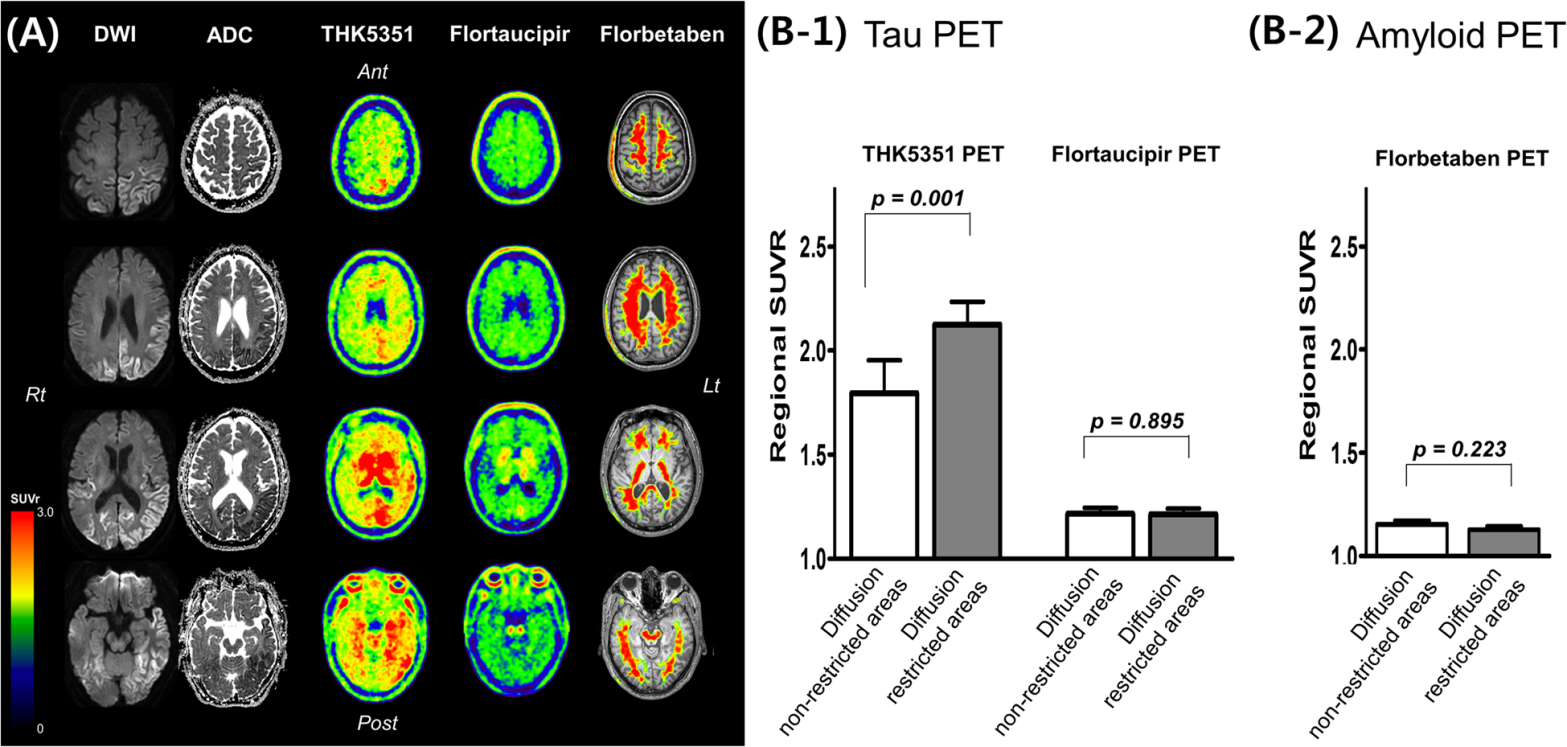
**a** Diffusion weighted images (DWI), apparent diffusion coefficient (ADC), tau (THK5351 and flortaucipir, and amyloid (florbetaben) PET images in a patient with sporadic Creutzfeldt-Jakob disease. **b** Regional standardized uptake value ratio (SUVR) of THK5351, flortaucipir, and florbetaben in diffusion non-restricted and restricted areas

CSF analysis showed that white blood cell count, red blood cell count, protein and glucose levels were all normal. However, 14–3-3 protein was weakly positive, total tau was highly elevated (1081.9 pg/ml, normal range 116–370 pg/ml), and phosphorylated tau was mildly elevated (87.0 pg/ml, normal range 35.84–66.26 pg/ml). Amyloid-ß was within the normal range (910.0 pg/ml, normal range 562–1018 pg/ml). Pathogenic prion protein (PrP^Sc^) was found using a RT-QuIC assay; however, the PRNP mutation was not found. Diffusion-weighted imaging (DWI) showed cortical diffusion restrictions in the left temporo-parieto-occipital regions (Fig. 1a). Based on his clinical symptoms and laboratory tests, the patient was diagnosed with probable sCJD.

The patient further underwent molecular PET imaging using ligands that bind to amyloid (^18^F-florbetaben) and PHF tau (^18^F-Flortaucipir and ^18^F-THK5351). ^18^F-florbetaben PET revealed amyloid negative. ^18^F-Flortaucipir PET showed focal uptake only in the left occipital white matter region. However, ^18^F-THK5351 PET showed diffuse high uptake on the left temporo-parieto-occipital regions, which largely overlapped with the diffusion restricted areas (Fig. 1a). To quantitatively measure tau uptake, we determined the voxel-wise ROI of diffusion restricted regions by hand-drawing using MRIcro (https://www.mccauslandcenter.sc.edu/crnl/). We calculated the standardized uptake value ratio (SUVR) of each THK5351, flortaucipir, and florbetaben uptake in diffusion restricted and unrestricted voxels. Then, to analyze whether the diffusion restricted area showed higher tau uptake, we parcellated the whole cerebral cortex into 84 regions based on an AAL template. We manually classified each parcellated region as a diffusion restricted area when more than 50% of the region showed diffusion restriction or as a diffusion non-restricted area when less than 50% of the region showed diffusion restriction. We then compared the regional SUVR of THK5351, flortaucipir, and florbetaben between diffusion restricted and non-restricted areas. We found that the mean THK5351 SUVR of diffusion restricted voxels was 2.17 whereas the mean THK5351 SUVR of diffusion non-restricted voxels was 1.79. The mean flortaucipir SUVR of diffusion restricted voxels was 1.16 whereas the mean flortaucipir SUVR of diffusion non-restricted voxels was 1.20. The mean forbetaben SUVR of diffusion restricted voxels was 1.07 whereas the mean florbetaben SUVR of diffusion non-restricted voxels was 1.16. Quantitative analyses showed that THK5351 standardized uptake value ratio (SUVR) in diffusion restricted areas was higher compared to diffusion non-restricted areas, while flortaucipir SUVR and florbetaben SUVR did not show any difference (Fig. 1b) (Additional file 1).

The patient died 13 months after his first visit and underwent brain autopsy. The time interval between imaging scans and autopsy was approximately 13 months (399 days for MRI, 396 days for florbetaben PET, 388 days for flortaucipir PET, and 378 days for THK5351 PET). Neuropathological analysis was performed at Chuncheon Sacred Heart Hospital, Chuncheon, Korea. Autopsies were performed according to the standard protocols of National Neuropathology Reference and Diagnostic Laboratories for Dementia (NRD) supported by Korea National Institute of Health [10, 11]. Neuropathological diagnostic analysis was performed on sections, including the frontal, occipital, and basal ganglia of the right and left hemisphere. For each immunohistochemical stain, the degree of pathology was graded as none, mild (< 10%), moderate (10–30%), or severe (> 30%).

**Fig. 2:**
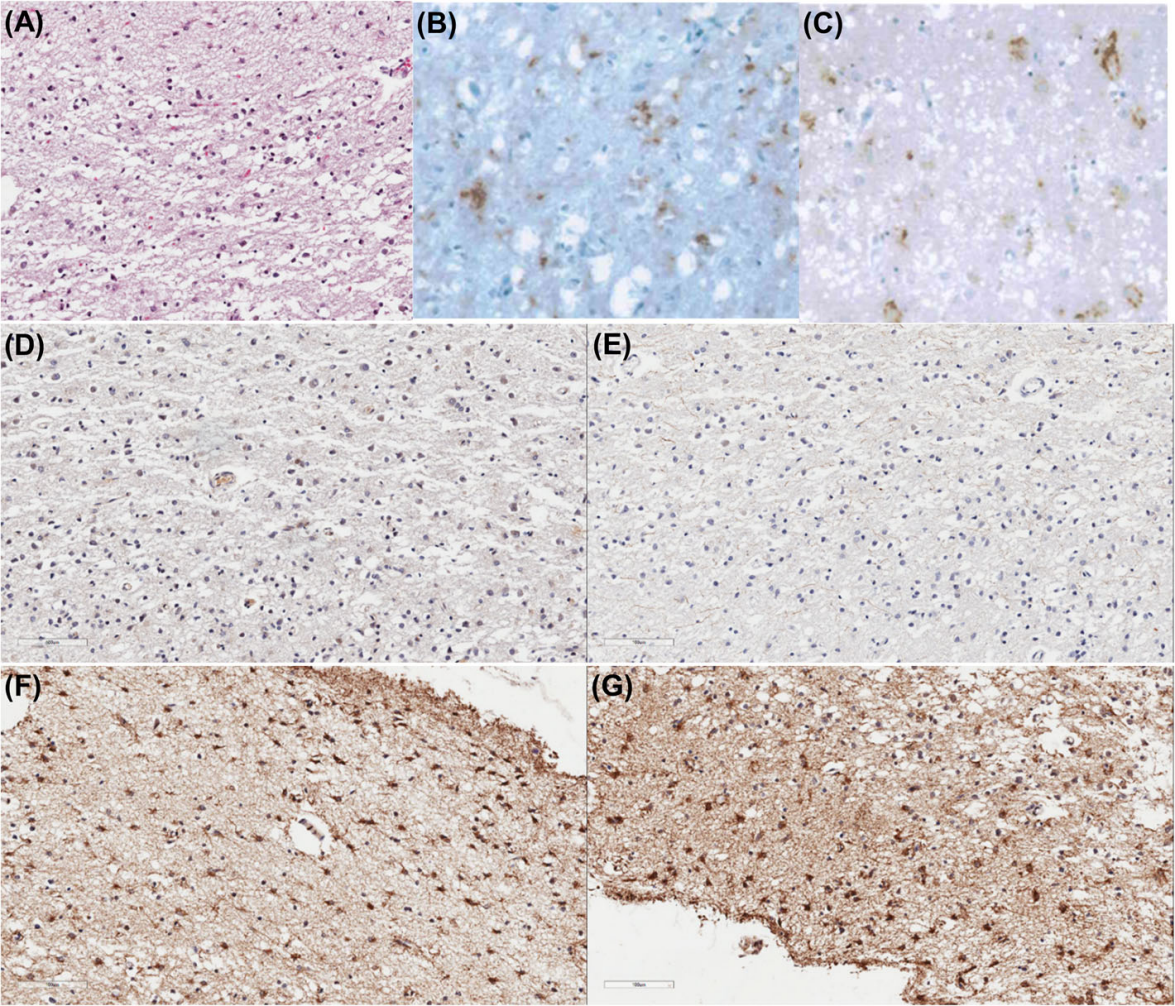
Pathological findings in the left occipital cortex of a patient with sporadic Creutzfeldt-Jakob disease. H&E staining (**a**) showed neuronal loss and vacuolation. Immunohistochemistry showed reactivity for PrP^sc^, **b** 3F4 antibody and **c** 1C5 antibody. Immunohistochemistry against amyloid-ß (**d**) and phosphorylated tau (**e**) showed no amyloid plaques and no neurofibrillary tangles, respectively. GFAP staining (**f**) showed active astrocytosis and MAO-B staining (**g**) showed increased MAO-B activity

A post-mortem study confirmed the diagnosis of CJD, as we found neuronal loss and micro-vacuolar degeneration on H&E (Fig. 2a) and immunoreactive for PrP^sc^ (3F4 and 1C5 antibody) (Fig. 2b-c). There was no evidence of neuritic plaques (Fig. 2d) or NFT (Fig. 2e) in the bilateral frontal, occipital cortices, and basal ganglia. However, we found mild diffuse amyloid plaques and mild neuropil threads. Glial fibrillary acidic protein (GFAP) stain showed the following results: moderate reactivity in the bilateral frontal and left occipital cortices; mild reactivity in the right occipital cortex and left basal ganglia; and non-reactivity in the right basal ganglia (Fig. 2f). MAO-B stain showed severe reactivity in the left frontal and bilateral occipital cortices and moderate reactivity in the right frontal cortex and bilateral basal ganglia (Fig. 2g).

## Discussion and conclusions

We report the PET findings of two tau ligands (flortaucipir and THK5351) and the autopsy results in a patient with sCJD. Our novel finding was that THK5351 uptake was increased in regions similar to diffusion restricted cortical areas, while flortaucipir uptake was not. The post-mortem study revealed no NFT but severe astrocytosis which was reactive for MAO-B staining. Therefore, our findings add pathological evidence that flortaucipir is more specific to PHF tau, and increased THK5351 uptake in sCJD might be an off-target binding driven by its high affinities to MAO-B.

These two tau ligands showed different uptake patterns in our patient with CJD. THK5351 uptake was increased in regions similar to diffusion restricted cortical areas, while flortaucipir uptake was not. Our finding is consistent with a recent study showing that CJD patients did not have any increased uptakes of flortaucipir [12]. Our imaging-pathological correlation study showed that there were mild neurophil threads but no NFT, suggesting that increased uptakes of THK5351 might represent off-target bindings. Indeed, previous studies showed that increased CSF tau in CJD is related to increased burdens of dystrophic neurites due to rapid destruction of neurons rather than development of PHF-type tau [7]. Our results are in line with our previous head to head comparison of the two ligands showing that flortaucipir is more sensitive and specific to PHF-type tau than THK5351 [4].

The underlying pathological substrate for THK5351 uptake in CJD might be related to increased MAO-B activity. We observed that GFAP staining of the region with diffusion restriction and high THK5351 uptake showed severe astrocytosis which was reactive for MAO-B staining. Our results are in line with previous reports showing sCJD patients have increased reactive astrocytes and MAO-B activity in the brain [5]. Previous studies suggested that THK5351 has high affinity for MAO-B, as ingestion of MAO-B inhibitor (selegiline) reduced THK5351 uptake [2], whereas MAO-B inhibitor did not block flortaucipir uptake in human brains [13]. Our findings, therefore, suggested that increased THK5351 uptake in this case might represent increased MAO-B activity within increased reactive astrocytosis.

Although THK5351 uptake regions largely overlapped with diffusion restricted areas, left frontal region showed discrepancy. The discrepancy between negative DWI and positive THK5351 uptake in the left frontal region might be explained by the difference in the underlying pathological substrate. Previous pathological studies showed that diffusion restriction on DWI correlated best with spongiform changes and PrP deposition, followed by reactive astrocytic gliosis [14, 15]. On the other hand, THK5351 uptake reflects increased MAO-B activity and increased reactive astrocytosis. Although autopsy findings in the left frontal region showed advanced features of sCJD (neuronal loss, micro-vacuolar degeneration, PrP^sc^ immunoreactivity, moderate reactivity on GFAP staining, and severe reactivity on MAO-B staining), we assume that at the time the patient underwent brain imaging, this region might have had astrocytosis with increased MAO-B activity but not spongiform changes or PrP deposition.

The limitation of this study is the 13-month delay between imaging scans and autopsy. As histopathological data were obtained at a more advanced stage than imaging data, vacuolation, PrPsc deposition, and reactive astrocytic gliosis with increased MAO-B activity were likely less severe at the time the patient underwent imaging. Therefore, DWI and THK5351 might not fully reflect the pathological findings. However, as there was no evidence of neuritic plaques or NFT even at the advanced stage, we conclude that THK5351 uptake in this sCJD patient represents off-target binding.

In conclusion, our imaging-pathological correlation study of THK5351 and flortaucipir suggested that flortaucipir is more specific to PHF tau, and increased THK5351 uptake in sCJD might be an off-target binding driven by its high affinities to MAO-B.

## Additional file

Additional file 1: Supplementary Methods. Detailed methods for PET acquisition and analysis. (DOCX 15 kb)

## Data Availability

The datasets used and/or analyzed during the current study are available from the corresponding author on reasonable request.

## Abbreviations

CSF: Cerebrospinal fluid
DWI: Diffusion-weighted imaging
GFAP: Glial fibrillary acidic protein
MAO-B: Monoamine oxidase B
NFT: Neurofibrillary tangles
PET: Positron emission tomography
PHF: Paired helical filament
PrP^Sc^: Pathogenic prion protein
sCJD: Sporadic Creutzfeldt-Jakob Disease
SUVR: standardized uptake value ratio
