# Supplementary material for: THK5351 and flortaucipir PET with pathological correlation in a Creutzfeldt-Jakob disease patient: a case report
Source: BMC Neurol. 2019 Aug 29;19:211. doi: 10.1186/s12883-019-1434-z (PMC6714095; doi:10.1186/s12883-019-1434-z)
Supplement: Supplementary file 1 — Supplementary Methods. Detailed methods for PET acquisition and analysis. (DOCX 15 kb) [file 12883_2019_1434_MOESM1_ESM.docx]

**THK5351 and Flortaucipir PET with pathological correlation in a Creutzfeldt-Jakob Disease patient: a case report**

**Supplementary Methods**

***^18^F-florbetaben PET acquisition***

Three days after the DWI was taken, the patient underwent ^18^F-florbetaben PET at Samsung Medical Center using a Discovery STE PET/CT scanner (GE Medical Systems, Milwaukee, WI, USA) in a three-dimensional scanning mode that examined 47 slices of 3.3 mm thickness spanning the entire brain. A 20-minute emission PET scan with dynamic mode (consisting of 4 x 5 min frames) was performed 90 minutes after injection of 318 MBq ^18^F-florbetaben.

***FTP PET acquisition***

Eleven days after the DWI was taken, the patient underwent FTP PET at Gangnam Severance Hospital using a Biograph mCT PET/CT scanner (Siemens Medical Solutions, Malvern, PA, USA). At 80 minutes after intravenous bolus injections of 389 MBq FTP, tau PET images were acquired for 20 minutes. Prior to the PET scan, we applied a head holder to minimise head motion and acquired brain CT images for attenuation correction. Three-dimensional PET images were reconstructed in a 256 × 256 × 223 matrix with a 1.591 × 1.591 × 1 mm voxel size using the OSEM algorithm (iteration=6 and subset=16).

***^18^F-THK5351 PET acquisition***

Ten days after the FTP PET was taken, the patient underwent THK5351 PET at Samsung Medical Center using a Discovery STE PET/CT scanner (GE Healthcare, Milwaukee, WI, USA). Tau PET images were acquired for 20 minutes, starting 50 minutes after an IV bolus injection of 177 MBq of ^18^F-THK5351. Using the ordered-subsets expectation maximisation algorithm (iteration=4 and subset=16), three-dimensional PET images were reconstructed in a 128 × 128 × 47 matrix with a 2.0 × 2.0 × 3.27 mm voxel size.

***PET data analysis***

PET images were co-registered to individual MRIs, which were normalised to a T1-weighted MRI template. We transformed the AAL template into the individual’s native T1 space using an inverse transformation matrix. Standardised uptake value ratios (SUVR) were calculated using the cerebellar grey matter in the native MRI space as a reference region. Data processing was performed using SPM version 8 (SPM8) through Matlab 2014b (MathWorks, Natick, MA, USA).
